# A Systematic Review and Meta-Analysis of Mindfulness-Based (Baduanjin) Exercise for Alleviating Musculoskeletal Pain and Improving Sleep Quality in People with Chronic Diseases

**DOI:** 10.3390/ijerph15020206

**Published:** 2018-01-25

**Authors:** Liye Zou, Albert Yeung, Xinfeng Quan, Sean David Boyden, Huiru Wang

**Affiliations:** 1 Department of Sports Science and Physical Education, The Chinese University of Hong Kong, Hong Kong, China; liyezou123@gmail.com; 2 Depression Clinical and Research Program, Massachusetts General Hospital, Harvard University, Boston, MA 02114, USA; AYEUNG@mgh.harvard.edu (A.Y.); SBOYDEN@partners.org (S.D.B.); 3 The South Cove Community Health Center, Boston, MA 02111, USA; 4 Department of Material Science and Engineering, Sichuan University-Pittsburgh Institute, Chengdu 610065, China; xinfeng.quan@gmail.com; 5 Department of Physical Education, Shanghai Jiaotong University, Shanghai 200240, China

**Keywords:** mind-body exercise, Baduanjin Qigong, pain, sleep quality

## Abstract

Objective: we performed the first systematic review with meta-analyses of the existing studies that examined mindfulness-based Baduanjin exercise for its therapeutic effects for individuals with musculoskeletal pain or insomnia. Methods: Both English- (PubMed, Web of Science, Elsevier, and Google Scholar) and Chinese-language (CNKI and Wangfang) electronic databases were used to search relevant articles. We used a modified PEDro scale to evaluate risk of bias across studies selected. All eligible RCTS were considered for meta-analysis. The standardized mean difference was calculated for the pooled effects to determine the magnitude of the Baduanjin intervention effect. For the moderator analysis, we performed subgroup meta-analysis for categorical variables and meta-regression for continuous variables. Results: The aggregated result has shown a significant benefit in favour of Baduanjin at alleviating musculoskeletal pain (SMD = −0.88, 95% CI −1.02 to −0.74, *p* < 0.001, *I*^2^ = 10.29%) and improving overall sleep quality (SMD = −0.48, 95% CI −0.95 to −0.01, *p* = 004, *I*^2^ = 84.42%). Conclusions: Mindfulness-based Baduanjin exercise may be effective for alleviating musculoskeletal pain and improving overall sleep quality in people with chronic illness. Large, well-designed RCTs are needed to confirm these findings.

## 1. Introduction

Musculoskeletal pain as a public health issue has been extensively investigated worldwide (e.g., in United States, Europe, Denmark, Singapore, Brazil, and Hong Kong) with respect to its prevalence [1,2,3,4,5,6]. Nine to forty percent of adults in the general population suffer from musculoskeletal pain (e.g., neck pain, shoulder pain, knee pain, low back pain, and widespread bodily pain), which was reported in these national-levels of epidemiological studies [1,2,3,4,5,6]. Of the adult population, individuals with chronic diseases (scapulohumeral periarthritis, ankylosing spondylitis, knee osteoarthritis, lumbar disc herniation, osteoporosis, and Type 2 diabetic mellitus) are more likely to have the complaints of these regional and widespread bodily pains [7,8,9,10]. Noteworthily, a high percentage of the symptomatic adults reported decreased work productivity, limited social participation, and poor sleep quality, ultimately leading to reduced quality of life [11]. According to the National Institute of Health [12], annual musculoskeletal pain-related expenses (e.g., ineffective treatment, over-the-counter medicine, medical doctor visits, the reduced number of working days, and decreased workability) had reached 65 billion US dollars; the economic burden of musculoskeletal pain is not only enormous for patients’ families, but also challenges the national healthcare system.

Given the enormous financial burden and reduced quality of life in these symptomatic adults, musculoskeletal pain management as a medical and public health issue has drawn attention from the research community [13,14,15,16]. Although considerable effort has been made to investigate the effects of pharmacological treatment on alleviating musculoskeletal pain, its side effects after prolonged use of the drug therapies subsequently persist and may last for a lifetime [17,18,19]. Physical therapy and manual therapy as mainstream western non-pharmacological treatments are commonly recognized in medical settings, but they require a significant amount of time and money at an authorized rehabilitation center [20,21,22,23]. People living in developing or underdeveloped countries who have no health insurance could not afford these costly treatments. Thus, readily accessible and cost-effective intervention requires no limitations of time and space that is urgently demanded.

Chinese traditional Health-Qigong exercises (CTHQ) (e.g., Taichi, Baduanjin, Liu Zijue, and Wuqinxi), characterized by an integration of slow coordinating postures and musculoskeletal stretching movements, meditative mind, and breathing techniques have been extensively studied with respect to the health benefits [24,25,26,27,28,29,30,31]. Particularly, a recently published randomized controlled trial indicated that Tai-chi as one of the CTHQ was as effective as physical therapy for musculoskeletal pain management in 204 patients with knee osteoarthritis [32]. As compared to Tai-Chi, Baduanjin is an easy-to-learn health-Qigong form that can be independently practiced at home or in the workplace, because it only contains eight movements [33]. More importantly, Baduanjin involves musculoskeletal stretching throughout the entire body while applying breathing regulation and an empty state of mind; it seems to be more reasonable that Baduanjin may have musculoskeletal pain-alleviating effects [34]. Sleep disturbance was reported to be associated with elevated activation of the autonomic nervous system [35]. To reduce the activation levels in the autonomous nervous system, sleep quality could be improved through relaxed state of mind and breathing regulation during Baduanjin practice [36]. Although a high number of experimental studies were conducted in recent years to substantiate the proposal [37,38,39,40,41,42], a systematic review with a meta-analytical method on this topic has not been done so far. Thus, we will systematically and critically evaluate the emerging research literature related to the effects of Baduanjin on musculoskeletal pain and sleep quality in people with chronic diseases. Additionally, we want to determine if Baduanjin practice over more extended periods of time is more effective than a short term of Baduanjin. If the musculoskeletal pain could be alleviated after Baduanjin intervention, we assume that overall sleep quality in these symptomatic individuals would be improved as well.

## 2. Methods

### 2.1. Search Strategy

A comprehensive literature search was conducted by a review author (FXQ) through 3 September to 12 September 2017. The English-language electronic databases (PubMed, Web of Science, Elsevier, and Google Scholar) were used for the literature search. In addition, we also used two well-respected Chinese academic databases; they are Chinese National Knowledge Infrastructure (CNKI) and Wanfang. The purpose of this systematic review was to investigate the effects of Baduanjin on musculoskeletal pain and sleep quality. To obtain a maximum number of eligible articles, the search terms (Baduanjin or Eight-section Brocade) were separately integrated with the two interesting outcomes: “Baduanjin and pain”, “Baduanjin and sleep quality”, “eight-section Brocade and Pain”, and “eight-section Brocade and sleep quality”.

### 2.2. Inclusion Criteria and Study Selection

To gain a comprehensive understanding of the effects of Baduanjin on musculoskeletal pain and sleep quality, all experimental studies (randomized controlled trial, RCT; non-randomized controlled study, NRCT; pretest-posttest study, PPS) were considered in this systematic review, but only RCT and NRCT were evaluated on the methodological quality, and their data were synthesized. Firstly, the experimental studies must be published in the peer-reviewed journals and full-text articles can be retrieved for data extraction. Secondly, each study must have no less than 10 adults who suffered at least one chronic disease (e.g., lower back pain, rheumatic disease, insomnia, Parkinson’s disease, diabetic mellitus, or hypertension). Thirdly, at least one of the two outcomes (musculoskeletal pain and sleep quality) must be reported in these studies. Fourthly, Baduanjin should be the primary exercise intervention (e.g., Baduanjin vs. no training, Baduanjin + drug therapy vs. drug therpy, Baduanjin + acupuncture vs. acupuncture, or Baduanjin + educational program vs. educational program). Exclusion criteria included review studies, observational studies, and conference abstracts. A review author independently identified all potential articles against the inclusion criteria, which was verified by a second review author (review all papers as well). Discrepancies between the two review authors were discussed to reach their agreement.

### 2.3. Assessment of Risk of Bias for Each Eligible Study

Risk of bias for selected RCT and NRCT was interpedently assessed by a review author using the modified Physiotherapy Evidence Database (PEDro) Scale, followed by a verification of another review author (review all studies selected as well). The original 11-item PEDro scale involves eligibility criteria (does not contribute to total score), randomization, concealed allocation, similar baseline, blinding of all participants, blinding of all therapists, blinding of all assessors, more than 85% retention, intention-to-treat analysis (if missing data is present), between-group comparison, and point measures and measures of variability. We removed blinding of participants and Baduanjin instructors, because these two items are impractical in exercise intervention. Additionally, given the fact that prior sample size calculation and isolated Baduanjin intervention could affect the internal validity, we incorporated them into the modified assessment tool. If a criterion is clearly satisfied, one point was awarded and vice versa. Because Item-1 (eligibility criteria) does not contribute to total score, a sum score ranging from zero to ten can be awarded for each study, but summary quality scores were not used to categorize studies selected in this systematic, as suggested by Costa, Hilfiker, and Egger [43].

### 2.4. Data Extraction and Synthesis

The same review authors independently performed data extraction using a pre-created summary Table 1. The data extracted are study characteristics (the name of the leading authors, year of publication, and type of experimental design), study location and language of publication, initial sample size, dropout rate, study participants (disease condition with reporting of mean age or age range), intervention program (training duration and dosage, total training hours, weekly training hours, and total training sessions), test administration (outcome measured and blinding of assessor), and adverse event and follow-up assessment. For the two interesting outcomes, mean (M) and standard deviation (SD) at baseline and post-intervention from each eligible RCT and NRCT were extracted when the number of participants in experimental and control groups was obtainable. We tried to contact the corresponding author of one study because summary statistics were not reported, but received no response (thus, we excluded it).

Meta-analytical methods were used to synthesize the study findings of all eligible individual studies examining the effects of Baduanjin on musculoskeletal pain and/or sleep quality. Comprehensive Meta-Analysis Version 2.0 software (BioStat, Englewood, NJ, USA) was used to compute effect size (standardized mean difference (SMD) reflects the magnitude of the Baduanjin intervention effect) and the 95% confidence intervals (CI) while random effects model was set. With respect to musculoskeletal pain or sleep quality, the heterogeneity of effect sizes across all eligible studies was determined based on the *Q*-value. The expected *Q*-value is equivalent to or less than the degree of freedom (the number of studies minus one), indicating that the homogeneity of effect sizes across studies exists. The value of *I*-squared was used to determine the ratio of true heterogeneity to total observed variation. Specifically, *I*-squared ranging from 0 to 100% can be categorized into low heterogeneity (25% or lower), moderate heterogeneity (25 to 50%), and high heterogeneity (75% to 100%). With respect to moderator analysis, we ran subgroup meta-analysis for categorical variables and meta-regression for continuous variables. In this systematic review, categorical variables include the final sample size of each eligible study (<60 vs. ≥60), type of control groups (active (drug therapy, manual therapy, or other behavioral program) vs. non-active (no training, waitlist, original lifestyle, or usual care)), Baduanjin intervention length (<12 weeks vs. ≥12 weeks), weekly training hours (<5 h per week vs. ≥5 h per week), and session length (less than or 45 min vs. >45 min). The number of total sessions and total hours of Baduanjin intervention is a continuous moderator. Additionally, publication bias was evaluated using the funnel plot and Egger’s regression.

## 3. Results

### 3.1. Literature Search

**Figure 1:**
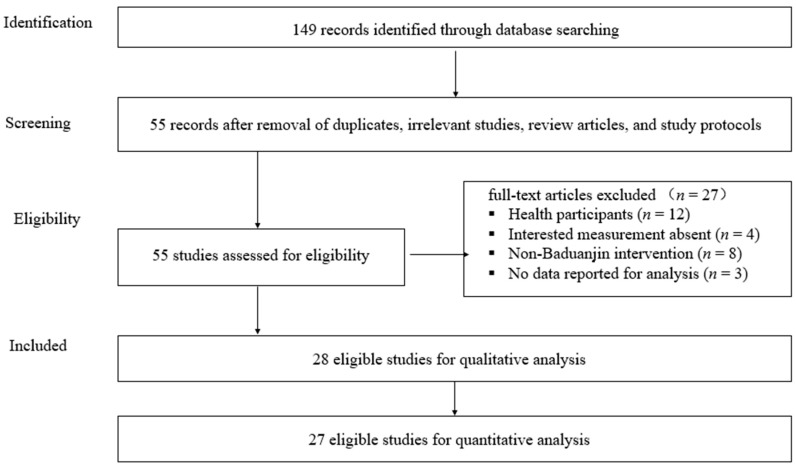
Flow of literature search and selection process.

Flow of literature search and selection process is shown in Figure 1. After both electronic and manual searches, 149 articles were initially identified. After exclusion of duplicates, irrelevant studies, review articles, and study protocols based on the titles and abstracts, 55 studies were left for further analysis. Of the remaining 55 studies, 27 full-text articles were excluded because of the following reasons: (1) healthy participants (with no diagnosed chronic conditions) (*n* = 12); (2) measurements of interest (pain and/or sleep quality) were not included (*n* = 4); (3) non-Baduanjin-based intervention (either incomplete Baduanjin used (*n* = 2) or other interventions were involved, e.g., medicine, manual therapy, or sports etc. (*n* = 6)); (4) incomplete data reported for analysis (*n* = 3). Our searches resulted in 28 eligible studies, including 25 RCTs, 2 NRCTs, and 1 PPS. When the similar baselines on the interesting outcomes were observed in the two NRCTs, we also considered them for meta-analysis in this study along with the 25 RCTs.

### 3.2. Study Characteristics

Table 1 shows the characteristics of the selected studies of 25 RCTs, 2 NRCTs, and 1 PPS, published between 2003 and 2017. Among the 28 studies, four were published in English, while the rest were in Chinese. All studies were carried out in mainland China except one, which was carried out in Hong Kong. Sample size of these studies ranged from 16 to 150 participants with 0 to 25% dropout rate. Collectively, this systematic review included a total of 1787 subjects with an age range of 15 to 80.

The disease conditions involve insomnia, body pain (shoulder, neck, and/or back), periarthritis, ankylosing spondylosis, lumbar disc herniation, osteoporosis, type 2 diabetic mellitus, radiculopathy, Parkinson’s disease, chronic fatigue syndrome-like illness, and hypertension accompanied with insomnia. Across all the 25 RCTs, the majority of the control groups received active interventions such as usual care, manual therapy, educational lessons, daily walking, drug therapy, acupuncture therapy (with or without cupping), and slinger exercise therapy, while one control group received no intervention and one control group waitlist. For the Baduanjin intervention groups, intervention times ranged from 2 weeks to 6 months, with 2 to 7 session per day in a week. A typical session lasted between 30 min to 90 min. The number of total sessions in an individual study ranged from 9 to 336, while the total session time ranged from 7 to 120 h. One study did not report detailed information on number of sessions and total time but did report the time span of the intervention. Most studies included professional or trained (but not reporting whether they had earned certifications) Baduanjin instructors to teach participants or lead the intervention. Most frequently used instruments for the measurement of pain were the Visual Analog Scale (VAS), followed by the Short-form McGill Pain Questionnaire (SF-MPQ), subscale (pain) of the Western Ontario and McMaster Universities Osteoarthritis Index (WOMAC), subscale (pain) of the Self-Rating Anxiety Scale (SAS), and the Japanese Orthopedic Association Back Pain Evaluation Questionnaire (JOABPEQ). Two studies did not report instrument name for pain measurement. For the sleep quality measurement, only one study adopted the Parkinson’s disease Sleep Scale, while the other 6 studies on sleep quality adopted the Pittsburgh Sleep Quality Index. Most studies reported no adverse events during the Baduanjin intervention, with 3 studies not mentioning adverse events. Only two studies reported follow-up assessment, 3 months and 6 months respectively, to track long term effect of Baduanjin on participants’ pain and/or sleep quality improvement.

### 3.3. Methodological Quality

The inter-rater agreement was high (95.4%) in selecting eligible studies. Methodological quality of all eligible studies was rated according to the modified PEDro scale, ranging from 4 to 8 points (Table 2). Of the 28 studies, none of them used the blinding of assessors and allocation concealment, which suggests fairly low quality. For the majority of the studies (*n* = 25, 89.3%), risk of bias also came from the combination of Baduanjin intervention and other components (e.g., drug therapy, manual therapy, acupuncture, and education program). The other major source of risk of bias resulted from the absence of a priori sample size estimation (*n* = 27, 96.4%).

### 3.4. Effects of Baduanjin on Musculoskeletal Pain and Overall Sleep Quality

#### 3.4.1. Baduanjin Intervention versus Control Group on Musculoskeletal Pain

**Figure 2:**
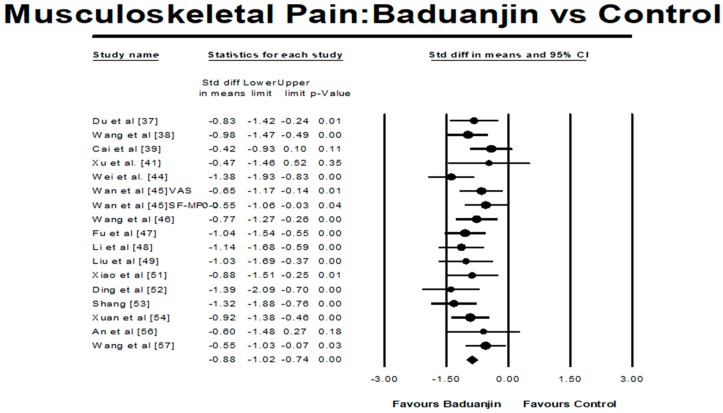
Effect of Baduanjin on musculoskeletal pain (Condition 1).

**Figure 3:**
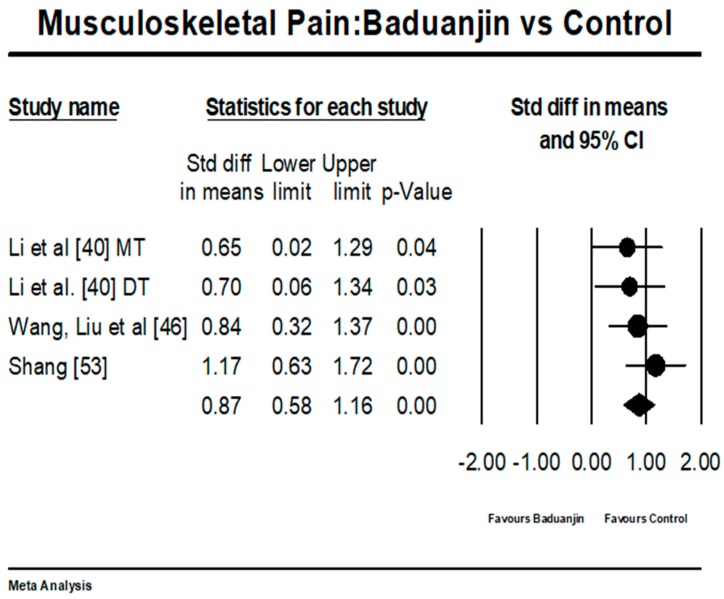
Effect of Baduanjin on musculoskeletal pain (Condition 2); MT = manual therapy; DT = drug therapy).

There were two types of testing instrument conditions for measuring musculoskeletal pain: (1) higher scores indicate more severe musculoskeletal pain (e.g., VAS and the SF-MPQ) as Condition 1; (2) higher scores indicate less pain (e.g., JOABPEQ) as Condition 2. For Condition one, there were 18 different studies (19 comparisons because one study by Wan et al. [45] used both VAS and SF-MPQ). The funnel plot was used to visually determine if the outliers existed: two studies (SMD = −2.75 and SMD = −2.37) reported large effect sizes beyond 2 SD above the mean effect size of −1.05 (random-effect model) [40,59]. While these two outliers were removed for further analysis, the funnel plot of remaining studies show no significant asymmetry (Egger’s regression intercept = −0.28, *p* = 0.85). For the mete-analysis, the 16 remaining studies (17 pairs of comparisons from 15 RCTs and 1 NRCT) examined the effects of Baduanjin versus control group on musculoskeletal pain. A higher negative value (effect size) indicates greater alleviation of musculoskeletal pain. The aggregated result has shown a significant benefit in favour of Baduanjin on alleviating musculoskeletal pain (a large effect size, but low heterogeneity: SMD = −0.88, 95% CI −1.02 to −0.74, *p* < 0.001, *I*^2^ = 10.29%; Figure 2). In the meta-analysis in Condition 2, there were three studies (4 pairs of comparisons); a higher positive value indicates greater alleviation of musculoskeletal pain. The aggregated result has shown a significant benefit in favour of Baduanjin on alleviating musculoskeletal pain (a large effect size without heterogeneity: SMD = 0.87, 95% CI 0.58 to 1.16, *p* < 0.001, *I*^2^ = 0%; Figure 3).

#### 3.4.2. Baduanjin Intervention versus Control Group on Overall Sleep Quality

**Figure 4:**
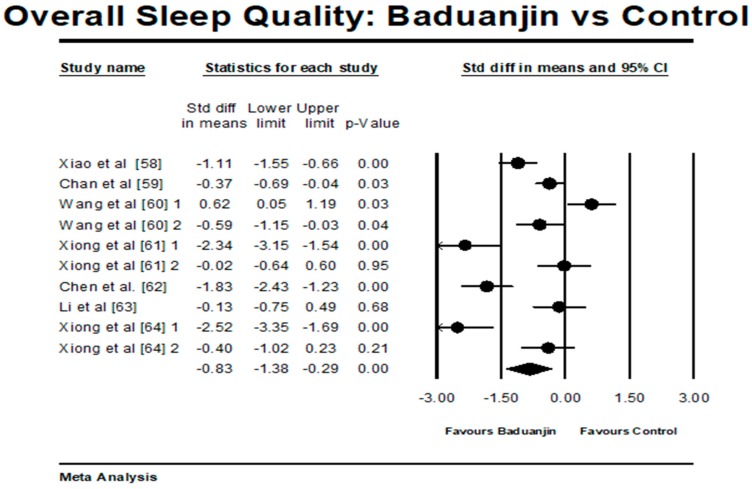
Effect of Baduanjin on overall sleep quality across all studies selected; Wang et al. [60] 1 = Baduanjin vs. LiuZijue; Wang et al. [60] 2 = Baduanjin vs. no training; Xiong et al. [61] 1 = Baduanjin + acupuncture vs. acupuncture; Xiong et al. [61] 2 = Baduanjin vs. acupuncture; Xiong et al. [64] 1 = Baduanjin + acupuncture vs. acupuncture; Xiong et al. [64] 2 = Baduanjin vs. acupuncture). 1 is referring to the first comparison, which is Baduanjin vs. Liuzijue; 2 for the second comparison in the same study.

**Figure 5:**
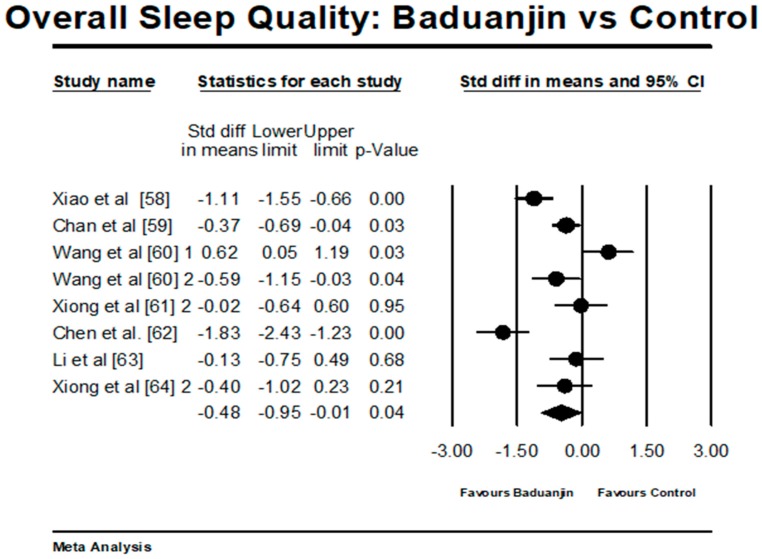
Effect of Baduanjin on overall sleep quality (without including two outliers); Wang et al. [60] 1 = Baduanjin vs. LiuZijue; Wang et al. [60] 2 = Baduanjin vs. no training; Xiong et al. [61] 2 = Baduanjin vs. acupuncture; Xiong et al. [64] 2 = Baduanjin vs. acupuncture). 1 is referring to the first comparison, which is Baduanjin vs. Liuzijue; 2 for the second comparison in the same study.

There were 7 studies (6 RCTs and 1 NRCT) investigating the effects of Baduanjin on overall sleep quality. Because three studies involved two control groups, ten pairs of comparisons were included for the meta-analysis [46,61,64]. A higher negative value (effect size) indicates better overall sleep quality. The aggregated result has shown a significant benefit in favour of Baduanjin on overall sleep quality (a large effect size, but high heterogeneity: SMD = −0.84, 95% CI −1.38 to −0.29, *p* = 0.002, *I*^2^ = 89.31%; Figure 4). The funnel plot was used to visually determine if the outliers existed: two pairs of comparisons (SMD = −2.52 and SMD = −2.34) reported large effect sizes beyond 2 SD above the mean effect size was −0.83 (random-effect model). While these two outliers were removed for further analysis, the funnel plot of remaining studies showing no significant asymmetry (Egger’s regression intercept = −0.376, *p* = 0.927). For the mete-analysis, the remaining comparisons examined the effects of Baduanjin versus control group on overall sleep quality. A higher negative value (effect size) indicates better overall sleep quality. The aggregated result has shown a significant benefit in favour of Baduanjin on alleviating overall sleep quality (a moderate effect size, but high heterogeneity: SMD = −0.48, 95% CI −0.95 to −0.01, *p* = 004, *I*^2^ = 84.42%; Figure 5).

#### 3.4.3. Moderator Analysis

The effects of potential moderator variables were computed only for musculoskeletal pain and not for overall sleep quality (the number of studies was less than 10). The results of categorical and continuous moderator analysis are presented in Table 3. With regard to the musculoskeletal pain, because the specific intervention length was not reported in five studies [45,50,51,54,55], only 13 studies were considered for subgroup analysis on the intervention length. With respect to the subgroup analysis for the rest of the categorical variables (final sample size, weekly training hours, sessions length, and control type), the 16 remaining studies (17 pairs of comparisons) were included. There were no significant effects for intervention length (*Q*(1) = 0.19, *p* = 0.66), final sample size (*Q*(1) = 0.07, *p* = 0.76), weekly training hours (*Q*(1) = 0.49, *p* = 0.49), session length (*Q*(1) = 0.49, *p* = 0.49), and type of control group (*Q*(1) = 0.21, *p* = 0.65). With regard to depression, there was a marginally insignificant effect for type of control group (*Q*(1) = 3.74, *p* = 0.053), whereas no significant effect was observed for intervention duration (*Q*(1) = 0.03, *p* = 0.87), training frequency (*Q*(1) = 2.8, *p* = 0.09), session length (*Q*(1) = 0.7, *p* = 0.4), and study quality (*Q*(1) = 0.44, *p* = 0.51). For continuous potential moderators, meta-regression indicated no significant effects for total hours in Baduanjin practice (β = −0.00111, 95% CI −0.0017 to −0.0039, *p* = 0.44) and the number of total sessions (β = −0.00108, 95% CI −0.00136 to 0.00352, *p* = 0.39) in terms of musculoskeletal pain. 

## 4. Discussion

This systematic review critically evaluated and statistically synthesized the evidence of the effects of Baduanjin exercise on musculoskeletal pain and sleep quality. Based on the available evidence, our review suggests that this traditional mind body exercise reduces musculoskeletal pain and improves sleep quality among patients with chronic illnesses. This is the first meta-analysis of the effects of Baduanjin on pain and sleep of patients with chronic illnesses. The data from the emerging literature provide support to Baduanjin as a self-management practice to augment conventional treatments for those with chronic illnesses. While the exact mechanisms of how Baduanjin affects musculoskeletal pain and sleep are unknown, the findings from this study are of great public health significance, since musculoskeletal pain and sleep disturbance are highly prevalent among these patients, and tremendous medical resources are used to address these symptoms. Contemporary concept Qigong practices like Baduanjin enhance physiological proprioception by combining a special state of awareness with posture, movement, and breathe control, and thereby improve and strengthen the overall state of vegetative regulation (homeostasis) [66]. Compared to first line treatments (drugs, cognitive behavioral therapy) and other effective treatment alternatives (e.g., aerobic exercise for insomnia), there are a lot of advantage to use Baduanjin exercise as an adjunctive treatment for patients suffering from pain and from insomnia. It is accessible to people of all ages and physical strength, easy to learn, and has few known side effects. This is particularly important for elderly patients who are prone to medication side effects and potential drug-drug interactions. The disadvantages are that qualified Qigong instructors may not be available in many areas, and that long term adherence of self-management practices tends to be low.

This study included recently published empirical studies in both English and Chinese that used Baduanjin as the primary intervention. This method is appropriate and important, since, thus far, all of the studies on Baduanjin were conducted in China (including Hong Kong) and were published mostly in the Chinese language. In including articles in Chinese, the contributions of researchers on Baduanjin studies published in Chinese peer-review journals are acknowledged, and the findings are more representative of the studies in this area. Other strengths of this study include the use of a standardized scale to assess the methodological quality of the studies, and a recognized meta-analytic method to evaluate the magnitude of Baduanjin intervention effect (pooled effect size), the variations in the frequency and duration of Baduanjin practice (moderator analyses), and the extent of asymmetry of effect sizes (funnel plot and Egger’s regression).

Nonetheless, the following methodological limitations could be acknowledged as they may influence the interpretation of these research findings. One of the most important drawbacks is that there was a lack of blinding in most studies, which might lead to subjectivity and social desirability bias. Second, Baduanjin was not offered as a monotherapy, but as an adjunctive treatment to the interventions received by the patients. It may be difficult to definitely conclude whether the outcomes were due to Baduanjin alone, to a synergetic intervention effect, or to the conventional treatment received by the patients. Nevertheless, our data provide support for Baduanjin as an adjunctive treatment for patients with chronic illnesses to reduce pain and improve sleep. Third, a variety of interventions were received by control groups, which made interpretations of outcomes difficult. Fourth, the duration of the Baduanjin practice varied a lot among different studies. This makes it hard to make specific recommendations about how frequent and how long practices should be. In addition, it is unclear why treatment effects did not differ by intervention length, frequency, and session length in moderator analysis. The intervention length of most studies was 4 weeks or more. It is possible that they have all attained the minimal duration needed to learn the Baduanjin postures and movements. The frequency and session length may be less important given that Baduanjin is relatively easy to learn, and that the majority of the benefits might have come from daily self-practice and not from the training sessions. Fifth, all the studies were conducted in China (mainland China and Hong Kong), and the participants were predominantly Chinese. It remains unclear whether the results are generalizable to non-Chinese populations. Sixth, a variety of heterogeneous chronic illnesses were included, consisting of insomnia, diabetes, hypertension, and various musculoskeletal conditions. It is not clear whether the beneficial effects of Baduanjin apply to all or only some of the chronic illnesses. Lastly, studies that report positive or significant results are more likely to be published, and outcomes that are statistically significant have greater possibility of being fully reported. Publication bias and outcome reporting bias might have existed in the included studies, and the effect sizes of Baduanjin might have been overestimated.

## 5. Conclusions

This systematic review, based on the existing literature, suggests that Bajuanjin may be an effective intervention to alleviate musculoskeletal pain and improve sleep quality among individuals with chronic illnesses. Significant methodological limitations were found in many of the empirical studies to date, which impacted the interpretation of these findings. More RCTs with rigorous research design are warranted to establish the therapeutic effects of Bajuanjin for musculoskeletal pain relief and improving sleep quality, and its potential to be used in part of healthy lifestyle intervention programs for populations with various clinical conditions.

## Figures and Tables

**Table 1:** Summary table relating to study.

**Author** **[Reference]**	**Location, Language**	**ISZ (BJ/CG)**	**Drop-Out %**	**Study Participants**		**Intervention Protocol**				**Test Administration**		**Adverse Event; Follow-Up**
				**Health Status**	**Mean Age or Age Range**	**Training Duration and Dosage**	**Total Hours**	**Hours Weekly**	**Total Session**	**Outcome Measured**	**Blinded Assessor**	
Du et al. [37], RCT	Qingdao, China (Chinese)	48 (24/24)	0%	People with Discogenic low back pain	age range from 38 to 56	BJ: 5 × 40 min/week for 8 weeks + usual care CG: usual care	27	3.33	40	Pain (VAS)	NR	No/No
Wang et al. [38], RCT	Hangzhou, China (Chinese)	76 (38/38)	5.3%	People with scapulohumeral periarthritis	Age range from 40 to 66	BJ: 5 × 60 min/week for 4 weeks + usual care CG: usual care	20	5	20	Pain (VAS)	NR	No/No
Cai et al. [39], RCT	Changsha, China (Chinese)	60 (30/30)	0%	patients with nerve-root type cervical spondylosis	A mean age of 50.4	BJ: 7 × 60 min/week for 6 months + usual care CG: usual care	168	7	168	Pain (VAS)	NR	No/No
Li et al. [40], RCT	Xi‘an, China (Chinese)	60 (20/20/20)	0%	Patients scapulohumeral periarthritis	A mean age of 50.43	BJ: 5 × 60 min/week for 6 months CG1: Twice per week (manual therapy) CG2: Drug therapy:	120	5	120	Pain (NR)	NR	No/No
Xu et al. [41], RCT	Suzhou, China (Chinese)	16 (8/8)	0%	Patients with lumbar disc herniation	A mean age of 48.1	BJ: 7 × 60 min/week for 1 month + drug therapy CG: drug therapy	28	7	28	Pain (VAS)	NR	No/No
Chen et al. [42], RCT	Chengdu, China (Chinese)	60 (30/30)	0%	People with osteoporosis suffered bodily pain	Age range from 55 to 71	BJ: 7 × 90 min/week for 8 weeks + usual care CG: usual care	84	10.5	56	Pain (VAS)	NR	No/No
Wei et al. [44], RCT	Liuzhou, China, (Chinese)	62 (31/31)	0%	People with ankylosing spondylitis	age range from 15 to 60	BJ: 7 × 40 min/week for 3 months + drug therapy (as same below) CG: drug therapy	56	4.67	84	Pain (VAS)	NR	No/No
Wan et al. [45], RCT	Shanghai, China (Chinese)	60 (30/30)	0%	People with myofascial pain syndrome on shoulder and neck	A mean age of 41.92	BJ: 7 × 60 min/week for 35 days + manual therapy (as same below) CG: 20 min manual therapy for 6 sessions	35	7	35	Pain (VAS and SF-MPQ)	NR	No/No
Wang et al. [46], RCT	Hangzhou, China, (Chinese)	67 (34/33)	3%	People with scapulohumeral periarthritis	Age range from 40 to 66	BJ: 5 × 60 min/week for 3 months + usual care CG: usual care	60	5	60	Pain (VAS)	NR	NR/No
Fu et al. [47], RCT	Langzhou, China (Chinese)	70 (35/35)	0%	People with ankylosing spondylitis	Age range from 17 to 42	BJ: 7 × 60 min/week for 6 months + drug therapy (as same below) CG: drug therapy	168	7	168	Pain (VAS)	NR	No/No
Li et al. [48], RCT	Jinan, China (Chinese)	60 (30/30)	0%	People with chronic low back pain	Age range from 35 to 60	BJ: 5 × 60 min/week for 8 weeks CG: Sling exerciser therapy	40	5	40	Pain (VAS)	NR	No/6-month
Liu et al. [49], RCT	Changsha, China (Chinese)	40 (20/20)	0%	People with Type 2 diabetic mellitus	A mean age of 56	BJ: 5 × 30 min/week for 6 months + drug therapy (as same below) CG: drug therapy	60	2.5	120	Pain (subscale of SAS)	NR	No/No
Wang, Liu et al. [50], RCT	Changsha, China (Chinese)	60 (30/30)	0%	People with scapulohumeral periarthritis	A mean age of 53.54	BJ: 7 × 60 min/week for 30 days + acupuncture (as same below) CG: acupuncture therapy	30	7	60	Pain (NR)	NR	No/No
Xiao et al. [51], RCT	Shanghai, China (Chinese)	44 (26/18)	0%	patients with cervical spondylotic radiculopathy/chronic neck pain	A mean age of 51	BJ: 7 × 60 min/week for 30 days + drug therapy CG: drug therapy	30	7	30	Pain (VAS)	NR	No/No
Ding et al. [52], RCT	Hefei, China (Chinese)	40 (22/18)	0%	people with chronic low back pain	A mean age of 60.98)	BJ: 5 × 40 min/week for 12 weeks CG: usual drug therapy (pain killers	40	3.33	60	Pain (VAS)	NR	No/No
Shang [53], RCT	Changchun, China (Chinese)	60 (30/30)	0%	People with lumbar disc herniation	Age range from 18 to 60	BJ: 5 × 60 min/week for 3 months + usual care CG: usual care	60	5	60	Pain (VAS and JOABPEQ)	NR	No/No
Xuan et al. [54], RCT	Shanghai, China, (Chinese)	80 (40/40)	0%	People with cervical spondylotic radiculopathy suffered from chronic neck pain	A mean age of 31.07	BJ: daily 40 min for 20 days + manual therapy (as same below) CG: manual therapy	13	4.67	20	Pain (SF-MPQ)	NR	No/No
Peng et al. [55], RCT	Guangzhou, China (Chinese)	100 (50/50)	9%	Older people with low back pain who suffered from osteoporosis	a mean age of 69.06	BJ: daily 30 min for 14 days + usual care CG: usual care	7	3.5	14	Pain (VAS)	NR	No/No
An et al. [56], RCT	Shanghai, China, (English)	28 (14/14)	25%	Female patients with knee osteoarthritis who suffered from bodily pain	a mean age of 65.0	BJ: 5 × 30 min/week for 8 weeks CG: no intervention	20	2.5	40	Pain (subscale of WOMAC)	NR	NR/No
Wang et al. [57] NRCT	Qingdao, China, (English)	72 (36/36)	4.2%	Patients with chronic neck pain	Age range 45 to 75	BJ: 7 × 30 min/week for 6 months + educational lessons CG: educational lessons	84	3.5	168	Pain (VAS)	NR	NR/No
Xiao et al. [58], RCT	Beijing, China (English)	96 (48/48)	7.3%	Patients with Parkinson’s disease	Age range from 55 to 80	BJ: 4 × 45 min/week for 6 months + daily walking for 30 min CG: daily walking for 30 min	72	3	96	Sleep quality (PDSS-2)	NR	No/No
Chan et al. [59], RCT	Hong Kong, China (English)	150 (75/75)	13.3%	People with chronic fatigue syndrome-like illness	A mean age of 39.1	BJ: 90 mn per session over 9 consecutive weeks, for 16 sessions CG: waitlist	24	2.6	16	Sleep quality (PSQI)	NR	No/3-month
Wang et al. [60], RCT	Beijing, China (Chinese)	90 (30/30/30)	13.3%	Patients with type 2 Diabetic mellitus accompanied by insomnia	a mean age of 57.8	BJ: training dosage was NR for 4 months + usual care CG1: LiuZijue (Training dosage was NR) for 4 months + usual care CG2: usual care	NA	NA	NA	Sleep quality (PSQI)	NR	No/No
Xiong et al. [61], RCT	Changchun, China, (Chinese)	60 (20/20/20)	0%	Middle-aged adults with insomnia	A mean age of 48.7	BJ1: 5 × 40 min/week for 4 weeks BJ2: Baduanjin (same dosage as above) + acupuncture CG: acupuncture	13.3	3.3	20	Sleep quality (PSQI)	NR	No/No
Chen et al. [62], NRCT	Fuzhou, China, (Chinese)	60 (30/30)	0%	Older people with hypertension accompanied by insomnia	Age range from 60 to 75	BJ: 3 × 60 min/week for 3 months + educational lessons CG: educational lessons	36	3	36	Sleep quality (PSQI)	NR	No/No
Li et al. [63], RCT	Jiangsu, China, (Chinese)	40 (20/20)	0%	Patients with type 2 Diabetic mellitus accompanied by insomnia (mean age of 53.6)	A mean age of 53.6	BJ: 7 × 30 min/week for 4 weeks + educational lessons CG: educational lessons	14	5.17	28	Sleep quality (PSQI)	NR	No/No
Xiong et al. [64], RCT	Changchun, China (Chinese)	60 (30/30)	0%	People with insomnia	Age range from 18 to 75	BJ1: 5 × 40 min/week for 4 weeks BJ2: Baduanjin (same dosage as above) + acupuncture CG: acupuncture	13.3	3.3	20	Sleep quality (PSQI)	NR	No/No
**Pretest-posttest study without control group**												
**Author** **Reference**	**Study location** **(Language)**	**Initial sample size**	**Drop-Out %**	**Study characteristic**		**Intervention protocol**				**Study findings**		
				**Health status**	**Age range or mean age**	**Training Duration and Dosage**	**Total Hours**	**Hours Weekly**	**Total Session**	**Outcome Measured**	**Study results**	
An et al. [65], PPS	Shanghai, China, (English)	28	21.4%	Patients with knee osteoarthritis	A mean age of 65.2	BJ: 5 × 30 min/week for 1 year	120	2.5	336	Pain (subscale of WOMAC)	BJ significantly reduced the level of pain (132.0 ± 69.6 vs. 56.2 ± 67.6, *p* = 0.000)	

Note: ISZ = initial sample size; AR = attribution rate; SAR = session attendance rate; BJ = Baduanjin; CG = control group; VAS = Visual Analogue Scale; SF-MPQ = the Short-form McGill Pain Questionnaire; SAS = self-rating Anxiety Scale; WOMAC = the Western Ontario and McMaster Universities Osteoarthritis Index; PDSS-2 = Parkinson’s Disease Sleep Scale; PSQI = Pittsburgh Sleep Quality Index; NPQ = the Northwick Park Neck Pain Questionnaire (NPQ); JOABPEQ = Japanese Orthopedic Association Back Pain Evaluation. NA = not applicable.

**Table 2:** Methodological quality for randomized controlled trials and non-randomized controlled studies.

Author (Reference)	Item 1	Item 2	Item 3	Item 4	Item 5	Item 6	Item 7	Item 8	Item 9	Item 10	Item 11	Score
Du et al. [37]	1	1	0	1	0	1	1	1	1	0	0	6/10
Wang et al. [38]	1	1	0	1	0	1	0	1	1	0	0	5/10
Cai et al. [39]	1	1	0	1	0	1	1	1	1	0	0	6/10
Li et al. [40]	1	1	0	1	0	1	1	1	1	0	0	6/10
Xu et al. [41]	1	1	0	1	0	1	1	0	1	0	0	5/10
Chen et al. [42]	1	1	0	1	0	1	1	1	1	0	0	6/10
Wei et al. [44]	1	1	0	1	0	1	1	1	1	0	0	6/10
Wan et al. [45]	1	1	0	1	0	1	1	1	1	0	0	6/10
Wang et al. [46]	1	1	0	1	0	1	0	1	1	0	0	5/10
Fu et al. [47]	1	1	0	1	0	1	1	1	1	0	0	6/10
Li et al. [48]	1	1	0	1	0	1	1	1	1	0	0	6/10
Liu et al. [49]	1	1	0	1	0	1	1	1	1	0	0	6/10
Wang & Liu [50]	1	1	0	1	0	1	1	1	1	0	0	6/10
Xiao et al. [51]	1	1	0	1	0	1	1	1	1	0	0	6/10
Ding et al. [52]	1	1	0	1	0	1	1	1	1	1	0	7/10
Shang [53]	1	1	0	1	0	1	1	1	1	0	0	6/10
Xuan et al. [54]	1	1	0	1	0	1	1	1	1	0	0	6/10
Peng et al. [55]	1	1	0	1	0	1	0	1	1	0	0	5/10
An et al. [56]	1	1	0	1	0	0	0	1	1	1	0	5/10
Wang et al. [57]	1	0	0	1	0	1	0	1	1	0	0	4/10
Xiao et al. [58]	1	1	0	1	0	1	1	1	1	0	0	6/10
Chan et al. [59]	1	1	0	1	0	1	1	1	1	1	1	8/10
Wang et al. [60]	1	1	0	1	0	1	1	1	1	0	0	6/10
Xiong et al. [61]	1	1	0	1	0	1	1	1	1	0	0	6/10
Chen et al. [62]	1	0	0	1	0	1	1	1	1	0	0	5/10
Li et al. [63]	1	1	0	1	0	1	1	0	1	0	0	5/10
Xiong et al. [64]	1	1	0	1	0	1	1	1	1	0	0	6/10

Note: Item 1 = eligibility criteria (does not contribute to total score); Item 2 = randomization; Item 3 = concealed allocation; Item 4 = similar baseline; Item 5 = blinding of assessors; Item 6 = more than 85% retention; Item 7 = missing data management (intention-to-treat analysis); Item 8 = between-group comparison; Item 9 = point measure and measures of variability; Item 10 = isolated Baduanjin intervention; Item 11 = prior sample size estimation; 1 = explicitly described and present in details; 0 = absent, inadequately described, or unclear.

**Table 3:** Moderator analysis for Baduanjin versus control group.

**Categorical Moderator**	**Outcome**	**Level**	**No. of Studies/Comparisons**	**SMD**	**95% Confidence Interval**	***I*^2^, %**	**Test for between-Group Homogeneity**		
							***Q*-Value**	**df(*Q*)**	***p*-Value**
Intervention Length	Pain	<12 weeks	4	−0.85	−1.24 to −0.46	0%	0.19	1	0.66
		≥12 weeks	9	−0.95	−1.17 to −0.74	39.03%			
Final sample size	pain	<60	6	−0.92	−1.22 to −0.61	0%	0.07	1	0.79
		≥60	11	−0.87	−1.04 to −0.70	30.78%			
Weekly training hours	Pain	<5 h per week	7	−0.95	−1.18 to −0.71	18.7%	0.49	1	0.49
		≥5 h per week	10	−0.84	−1.03 to −0.65	9.41%			
Session length	Pain	Less than 45 min	7	−0.95	−1.18 to −0.71	18.7%	0.49	1	0.49
		45 min or longer	10	−0.84	−1.03 to −0.65	9.41%			
Control type	Pain	Active	11	−0.91	−1.09 to −0.72	12.78%	0.21	1	0.65
		passive	6	−0.83	−1.08 to −0.59	18.63%			
**Continuous moderator**		**Level**	**No. of Studies/Comparisons**	β	**95% Confidence Interval**		***Q*-Value**	**df(*Q*)**	***p*-Value**
Total training hours		Pain	17	−0.00111	−0.0017 to 0.0039		0.6	1	0.44
Number of total sessions		Pain	17	−0.00108	−0.00136 to 0.00352		0.75	1	0.39
